# Gene Network Dysregulation in Dorsolateral Prefrontal Cortex Neurons of Humans with Cocaine Use Disorder

**DOI:** 10.1038/s41598-017-05720-3

**Published:** 2017-07-14

**Authors:** Efrain A. Ribeiro, Joseph R. Scarpa, Susanna P. Garamszegi, Andrew Kasarskis, Deborah C. Mash, Eric J. Nestler

**Affiliations:** 1 Fishberg Department of Neuroscience, Friedman Brain Institute, Icahn School of Medicine at Mount Sinai, New York, NY USA; 2 Department of Genetics and Genomic Sciences, Icahn Institute for Genomics and Multiscale Biology, Icahn School of Medicine at Mount Sinai, New York, NY USA; 3 Department of Neurology, University of Miami Miller School of Medicine, Miami, Florida USA

## Abstract

Metabolic and functional alterations of neurons in the dorsolateral prefrontal cortex (dlPFC) are thought to contribute to impulsivity, which is a hallmark of addictive behaviors that underlie compulsive drug seeking and taking in humans. To determine if there is a transcriptional signature in dlPFC neurons of humans with cocaine use disorder, we performed total RNA-sequencing on neuronal nuclei isolated from post-mortem dlPFC of cocaine addicts and healthy controls. Our results point toward a transcriptional mechanism whereby cocaine alters specific gene networks in dlPFC neurons. In particular, we identified an AP-1 regulated transcriptional network in dlPFC neurons associated with cocaine use disorder that contains several differentially expressed hub genes. Several of these hub genes are GWAS hits for traits that might involve dysfunction of brain reward circuitry (Body-Mass Index, Obesity) or dlPFC (Bipolar disorder, Schizophrenia). Further study is warranted to determine their potential pathophysiological role in cocaine addiction.

## Introduction

Early studies identified the prefrontal cortex (PFC) as a brain region that undergoes significant changes after long-term cocaine use. For example, using brain imaging techniques it has been shown that long-term cocaine users have reduced volume of PFC, which is accompanied by functional hypoactivity in the region^1–4^. As a highly evolved portion of frontal cortex, PFC is currently thought to mediate inhibitory control over behavior as a normal brain process^5^. Therefore, dysfunction of this brain region is thought to lead to impulsivity, which is a hallmark of addictive behaviors that underlie compulsive drug seeking and taking^6–8^. Numerous studies have sought to translate this clinical work by showing that PFC dysfunction in rodents leads to a loss of inhibitory control and increased drug seeking behaviors in animal models^9–11^. While the homology between rodent and human PFC remains uncertain, some functional studies suggest that human dorsolateral PFC (dlPFC), or Broadman’s Area 46, may play a similar role to medial PFC in rodents^12–15^. However, it is clear that rodent mPFC does not account for all of the diverse functions of human dlPFC, leading to the view that it first evolved in non-human primates^16^.

While there is a large literature concerning changes in the function, morphology, and metabolism of the human PFC after chronic cocaine use, little is known about the transcriptional alterations that underlie these changes in human dlPFC, which has been suggested as a target for treating addiction^17^. An earlier study performed gene expression profiling using microarrays in brain samples of human cocaine abusers^18^. One limitation of this study is that it used whole tissue extracts for expression profiling, leading to their identification of prominent changes in oligodendrocytic transcripts in their analysis. The presence of multiple cell types in brain tissue samples thus prevents detection of neural-specific pathophysiology. Another limitation is the reliance on microarray technology, which has many disadvantages when compared to next generation sequencing technologies. There has also been little to no progress in the discovery of novel drugs for the treatment of cocaine use disorder. A gap thus remains in our understanding of how transcription in dlPFC neurons is altered after cocaine use in humans, and how those neuroplastic changes relate to abnormal functioning of the region.

To address this, we performed total RNA-sequencing on fluorescent-activated cell sorting (FACS)-isolated dlPFC neuronal nuclei from humans who were chronic users of cocaine with severe patterns of use and from healthy controls. Cocaine intoxication deaths were selected based on forensic autopsy certification of the cause and manner of death^19^. All cases selected for analysis met criteria for cocaine dependence with intoxication at autopsy (ICD-10 F14.22) and DSM-IV diagnostic criteria for cocaine abuse or dependence (replaced by cocaine use disorder in DSM-5). Cocaine users are at high risk for developing cocaine use disorder^20–22^. The cocaine cases selected for this study were chronic users, many of whom were “crack” cocaine users based on informant interviews and scene investigations.

After we validated the cell type-specificity of our approach, we identified 883 differentially expressed transcripts in dlPFC neurons of cocaine addicts, several of which have known roles in neuroplasticity underlying drug addiction. We then performed Weighted Gene Co-Expression Network Analysis (WGCNA) and identified a gene network in dlPFC neurons whose expression is altered in cocaine users compared to healthy controls. Several of the differentially expressed hub genes in this module are GWAS hits in other diseases with potential neuropsychiatric components. Our results corroborate previous studies identifying increased AP-1 mediated transcription in the brain after cocaine administration and provide an important translational step forward by showing these same signaling pathways are altered in neurons of human addicts.

## Results

### Cell type-specific total RNA-sequencing from human post-mortem dlPFC neurons

We performed total RNA-sequencing on dlPFC neuronal nuclei isolated from humans with chronic cocaine use disorder who died from cocaine intoxication and from healthy controls (Table 1) (Supplementary Table 1). The cocaine cases were sampled from a robust collection of brains taken at forensic autopsy. We utilized a method for obtaining total RNA from neurons in frozen human brain tissue that does not require ribosomal depletion based on previous methods for isolating neuronal nuclei from human post-mortem brains^23–25^. Importantly, the nuclear sorting method we used minimizes aberrant transcription induced during FACS when compared to sorting methods that rely on enzymatic dissociation of brain tissue^24^. We did not observe any difference in the abundance of neuronal nuclei across cases and controls, suggesting that any brain volume loss observed in the dlPFC of human cocaine addicts is not due to loss of neurons (Table 1). Likewise, there was no difference in the average RIN (RNA integrity number) values of isolated RNA between cases and controls; RIN values are low—compared to those seen for whole cell RNA extracts—due to the lack of rRNA content in isolated nuclei. The high quality of the extracted nuclear RNA was nonetheless confirmed by the broad distribution of transcript sizes (Supplementary Figure 4).

**Table 1:** Cohort metadata.

	Healthy control	Cocaine intoxication death
Age	36.88 +/− 3.31	33.26 +/− 1.83
PMI	18.39 +/− 1.55	14.88 +/− 1.37
Race	AA (7), H (5), C (4)	AA (7), H (7), C (5)
% NeuN (+)/gated nuclei	36.36 +/− 1.96	31.57 +/− 2.60
% rRNA (+)	0.88 +/− 0.49	0.55 +/− 0.25
Total reads per sample	106,415,431 +/− 15,930,326	100,325,045 +/− 10,064,111

All cases were male. Mean +/− SEM are shown. (t-test with Welch’s correction: RIN: Health control (n = 17) 2.747 +/− 0.1258 vs. Cocaine intoxication death (n = 19) 3.005 +/− 0.2160, p > 0.05; Age: 36.88 +/− 3.319 vs. 33.26 +/− 1.837, p > 0.05) (student’s t-test: PMI: Healthy control (n = 17) 18.39 +/− 1.554 vs. Cocaine related (n = 19) 14.88 +/− 1.377, p > 0.05; % NeuN/Gated nuclei: Healthy control 36.36 +/− 1.96 vs. 31.57 +/− 2.60, p > 0.05).

**Figure 1:**
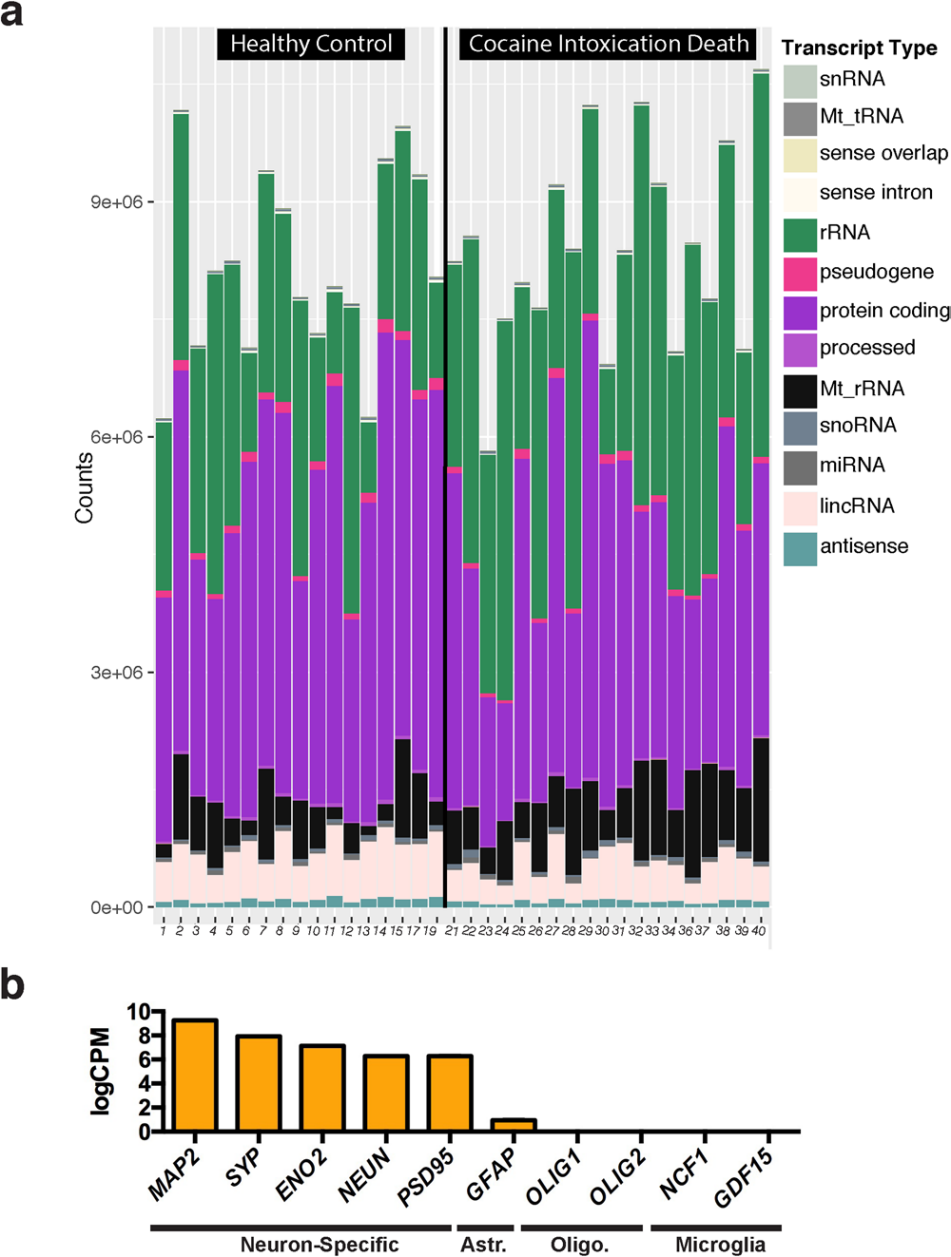
Cell type-specific total RNA-sequencing in human post-mortem dlPFC neuronal nuclei. (**a**) Quantification of read counts for each transcript type that was detected using RNA-sequencing. Healthy controls are shown on the left while cocaine cases are on the right. We did not detect any statistically significant differences in abundance of transcript types across cases and controls. (**b**) Neuron-specific transcripts are highly expressed, including microtuble associated protein 2 (*MAP2*), synaptophysin (*SYP*), enolase 2 (*ENO2*), neuronal nuclear protein (*NEUN*, also known as *RBFOX3*) and postsynaptic density 95 (*PSD95*). The astrocyte specific protein, glial fibrillary acidic protein (*GFAP*) was expressed at several orders of magnitude lower levels, while microglial and oligodendrocyte specific transcripts (*OLIG1*/2, oligodendrocyte transcription factor 1/2; *NCF1*, neutrophil cytosolic factor 1; and *GDF15*, growth differentiation factor 15) were not detected. Y-axis is log counts Per million. Standard error of the mean is shown. Astr. – Astrocyte-specific, Oilgo. – Oligodendrocyte-specific.

After sequencing, we compared the relative abundance of transcript types across all samples and found that we captured a comparable amount of each type across all samples (Fig. 1a). We did not observe any differences in relative transcript type abundance across cocaine and control cases. These data show that we consistently captured all transcript types in RNA-sequencing from human post-mortem neurons in an unbiased manner. As we did not perform a ribosomal depletion step, we also captured a wide variety of neuron-specific structural and regulatory RNAs that are otherwise lost with traditional poly-A-selection library preparation approaches for RNA-sequencing. Many of these transcripts are annotated but do not have known functions. Importantly, the rRNA in our samples did not saturate the reads in any sample, and we reliably quantified protein-coding transcript expression across all samples. The total transcriptome is available as a resource in Supplementary Table 2.

Confirming the cell type-specificity of our sequencing data, we observed high expression (>7logCPM) of several neuron-specific transcripts such as: microtubule associated protein 2 (*MAP2*), synaptophysin (*SYP*), enolase 2 (*ENO2*), neuronal nuclear protein (*NEUN*), and postsynaptic density 95 (*PSD95*) (Fig. 1b). Glial fibrillary acidic protein (*GFAP*), a marker for astrocytes, is barely present—at several orders of magnitude lower levels, while markers from oligodendrocytes and microglia were not detected^26, 27^. These data validate our FACS sorting method for isolating neuronal nuclei from human post-mortem brain tissue and verify the purity of our total RNA-sequencing samples.

### Transcriptional alterations in human post-mortem dlPFC neurons of cocaine-addicted individuals

**Figure 2:**
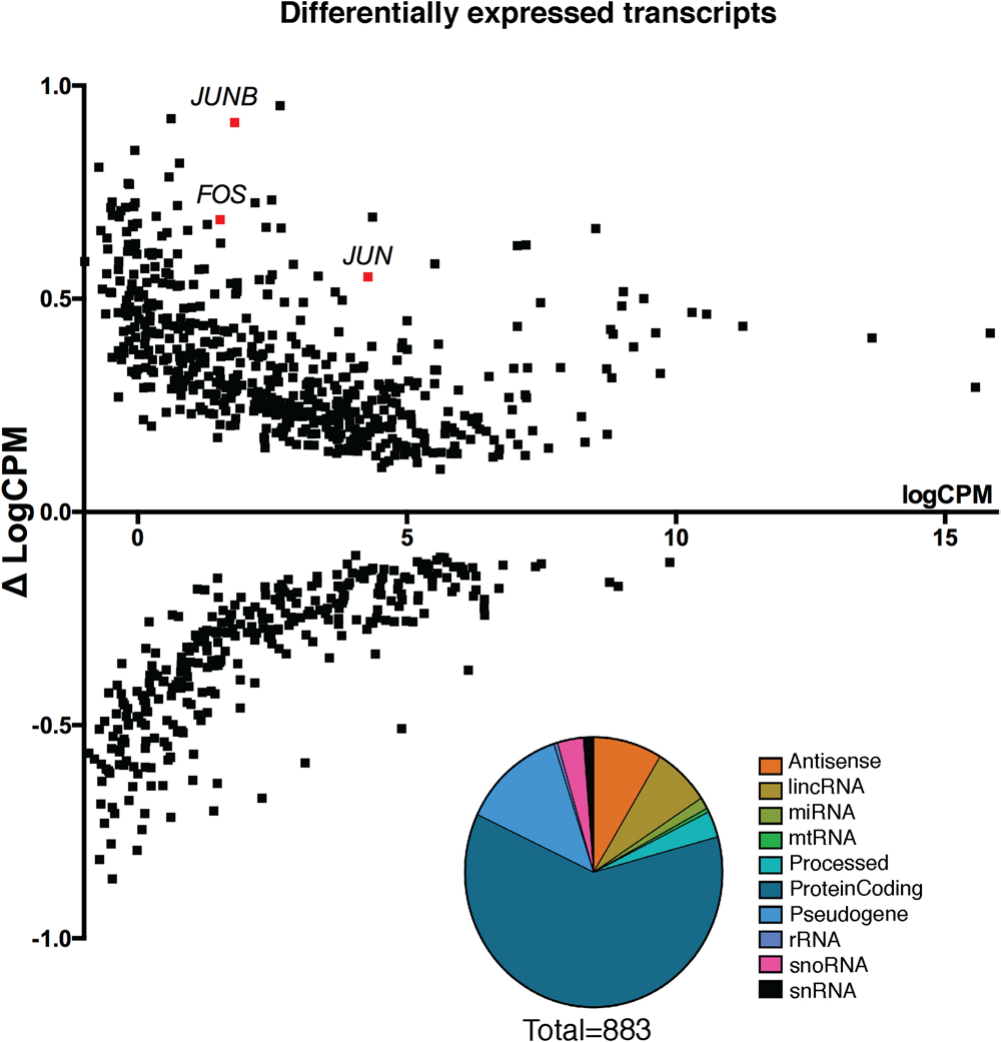
Differential expression of transcripts in human dlPFC between cocaine and control cases. We used Voom limma to identify 883 transcripts with a nominal p-value < 0.05. We observed up-regulation of *c-FOS* and *c-JUN* transcription factors, shown in red, which are immediate early genes known to play a role in transcriptional alterations underlying neuronal plasticity. (*c-FOS*: p < 0.02; *c-JUN*: p < 0.01; *JUND*: p < 0.01). Y-axis is delta of logCPM = ([logCPM Avg. Cocaine] − [logCPM Avg. Control]) and denotes up- or down-regulation of transcription. (Inset) Quantification of types of 883 transcripts with p < 0.05.

After validating the cell type-specificity and purity of our RNA-sequencing, we performed a differential expression analysis and identified transcriptome-wide alterations in transcript expression induced by cocaine use disorder in dlPFC neurons. Our data identify several up-regulated transcripts that have been implicated previously as general mechanisms underlying drug-induced neuronal plasticity, including *c-JUN*, *c-FOS*, and *JUNB* (Fig. 2)^28–30^. Our data support the literature identifying these transcription factors as mediators of transcriptional alterations seen in brain in addiction via the AP-1 binding site^31^.

Overall, our differential expression signature contains 10 different transcript types across 883 transcripts. We quantified the proportion of transcript types in our differential expression signature and found that more than half of the alterations identified were in protein-coding transcripts, showing that our total nuclear RNA-sequencing strategy robustly captures transcriptional activity of protein-coding genes. Alterations in pseudogene transcripts were the second most abundant, followed by alterations in lincRNAs (long intervening non-coding RNAs) and antisense transcripts. We note that non-coding RNA transcripts as a whole were overrepresented in the differentially expressed transcripts (p < 0.0001, Odds Ratio = 1.3[1.1–1.5]) while protein-coding transcripts were not (p > 0.05), suggesting that non-coding RNAs were significantly and preferentially altered. The enrichment of non-coding RNAs in the differential expression signature compared to their abundance in the genome highlights the advantage of using total RNA-sequencing in human samples to identify transcriptional mechanisms of disease pathology, as non-coding transcripts have been shown to play a critical role in regulating post-transcriptional processing of their functional homologs and remain an elusive target in our understanding of human disease^32^.

### Calculating gene co-expression networks in dlPFC neurons of cocaine-addicted humans

**Figure 3:**
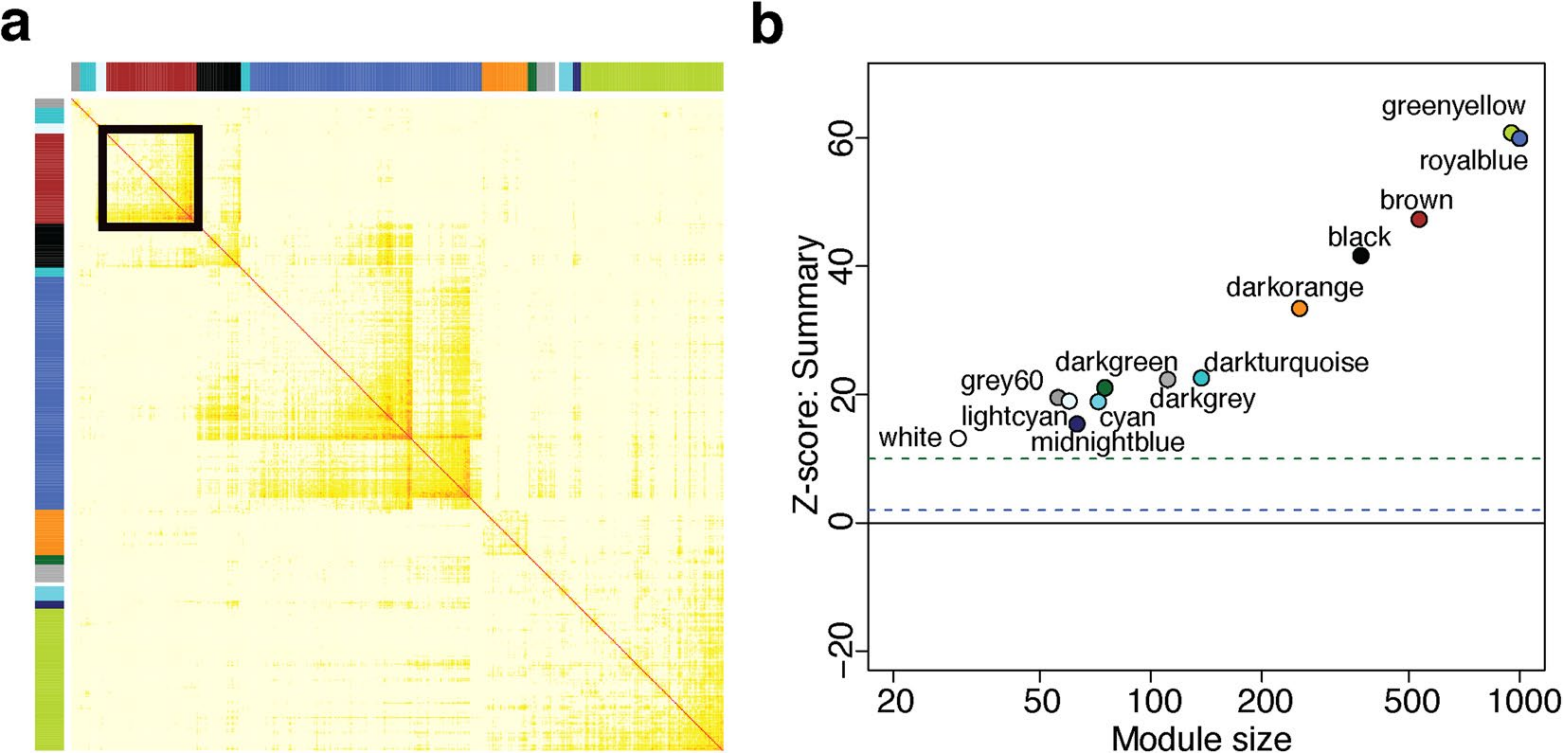
Weighted Gene Co-expression Network Analysis (WGCNA) of total RNA-sequencing data from human dlPFC neurons from cocaine and control cases reveals 13 distinct co-expression modules. (**a**) Each module is labeled by an arbitrary color represented at top and at left, and darker yellow and red represent greater topological overlap. (**b**) Z < 2 (blue line) suggest there is weak evidence for module robustness, while Z > 10 (green line) denotes strong evidence for their reproducibility. Evidence is considered moderate when 10 > Z > 2. All modules identified in dlPFC have Z quality scores > 10 (green line), suggesting there is strong evidence for their robustness and reproducibility.

To study dlPFC transcriptional networks associated with cocaine use disorder, we calculated weighted gene co-expression networks on the total transcriptome of dlPFC neurons from all samples. We identified 13 distinct gene co-expression modules (Fig. 3a). Each module is comprised of a group of coexpressed genes—exhibiting strongly correlated patterns of expression in our dataset—and given an arbitrary color for a name. Resampling methods confirmed that these modules are robust and reproducible, and GO and KEGG (Kyoto Encyclopedia of Genes and Genomes) term enrichment suggests that these modules are functionally coherent and biologically meaningful (Fig. 3b). For example, we identified modules associated with mitochondrial metabolism (lightcyan), cell junctions (black), and mRNA splicing (darkturquoise), among others. Module membership and intramodular statistics are provided in Supplementary Table 2.

### Transcripts in the brown module are significantly enriched for several distinct biological processes related to neuroplasticity

**Figure 4:**
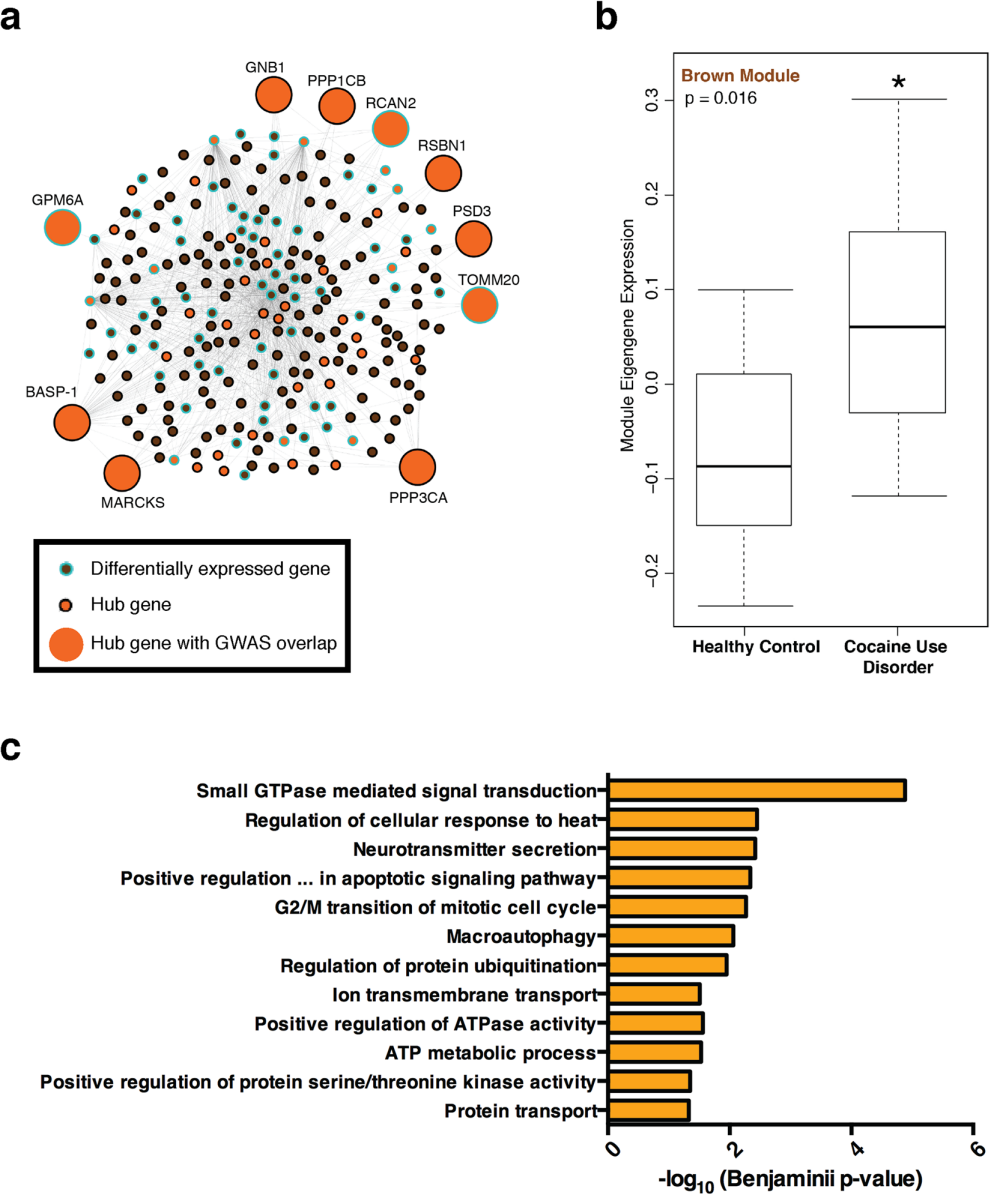
Brown module expression is increased in human dlPFC neurons from cocaine addicts. (**a**) Network diagram of brown module containing 550 transcripts, each shown as an individual node. Lines between nodes connect highly co-expressed transcripts within our dataset (threshold: k > 3). Orange nodes show Hub genes, defined as the top 10% most connected transcripts. Differentially-expressed transcripts are outlined in blue (p < 0.05). The larger nodes highlight differentially expressed hub genes that are hits in genome-wide association studies (GWAS) for several neuropsychiatric disorders (see text). (**b**) Mean eigengene expression is significantly increased in dlPFC neurons from cocaine cases compared to controls (Kruskal-Wallis test: p < 0.02). (**c**) Transcripts in the brown module are significantly enriched for several distinct biological processes related to neuroplasticity. We used the DAVID bioinformatics resource to identify biological processes enriched in the brown module. Each bar denotes the −log_10_ Benjamini p-values a specific Gene Ontology term. All biological process terms that achieved significance (benjamini p < 0.05) are shown.

To identify modules associated with case/control status, we first investigated if any modules were overrepresented with differentially expressed genes. We found that only 3 modules showed significant enrichment after Bonferroni correction: lightcyan (*P* = 2.4 × 10^−32^, Odds Ratio = 34.6[20–60–61]), grey60 (*P* = 8.4 × 10^−17^, Odds Ratio = 17[9.5–30]), and brown (*P* = 6 × 10^−34^, Odds Ratio = 5.6[4.4–7.1]). To investigate if these three modules were correlated with case/control status at the network level, we calculated each module’s eigengene, which approximates the average expression of genes in the module. We then correlated each module eigengene to case/control status. The brown module showed the strongest effect (*P* = 0.016), suggesting that it is the strongest candidate for downstream analysis of cocaine-related changes. The brown module includes 550 transcripts and exhibits higher module eigengene expression in the cocaine cohort, thus associating perturbations of this network with cocaine use disorder (Fig. 4b). Furthermore, it was significantly enriched for several biological processes including small GTPase signaling, neurotransmitter secretion, and regulation of ATP-related and metabolic processes suggesting that this network may play an important role in neuroplasticity (Fig. 4c). These analyses provide important validation that the brown module likely contains genes that contribute importantly to the aberrant patterns of gene expression seen in cocaine users.

### Identifying transcriptional regulators of the cocaine-associated gene module

To identify the most important genes in the brown module, we calculated connectivity measures for each gene in the network and identified the most highly connected (i.e., hub) nodes. The brown module has 53 hub genes, including genes associated with dopaminergic signaling (*GNB1*, *GSK3B*), long-term potentiation (*PPP1CB*), opioid signaling (*PPP2CA*, *PPP3CA*, *PPP3R1*), epilepsy (*SYN1*), and Parkinson’s disease (*SNCA*). Fourteen of the 53 hub genes were upregulated in the cocaine cohort (P = 1.5 × 10^−8^, Odds Ratio = 8.6[4.3–16.4]), suggesting that these hubs mediate cocaine modulation of this network.

Genes are often coexpressed because they share a common upstream regulator. To determine the upstream regulators that control expression of genes within the brown module, we used the Enrichr platform to investigate transcription factors known to bind to these genes. We found that *c-JUN* transcription factor targets are significantly enriched among our brown module hub genes using two independent databases of published chromatin immunoprecipitation sequencing (ChIP-seq) data (ChEA 2016: Jun_26020271 – corrected p value = 0.0045; Encode TF Chip-seq 2015: Jun – corrected p value = 0.0057). This analysis provides biological evidence using previously published ChIP data showing that *c-JUN*, an AP-1 transcription factor that is significantly upregulated in our dataset, is a primary regulator of brown module genes.

To validate that *c-JUN* controls the expression of the brown module, we analyzed previously published data that measured transcriptional changes in PC3 cells in response to *c-JUN* shRNA knockdown^33^. Such knockdown of *c-JUN* strongly affects the expression of brown module genes in PC3 cells (*P* = 0.01, OR = 1.6[1.1–2.2]), validating our prediction *in silico* that *c-JUN* is an upstream regulator of this module. This dataset focused specifically on AP-1 binding sequence containing genes, which are targeted by *FOS* and *JUN* family proteins, and suggests that *c-JUN* expression is required for AP-1 mediated transcriptional alterations induced by cocaine. Importantly, our human neuron-specific data validate and complement multiple decades of research that have followed the observation that chronic cocaine induces long-lasting AP-1 complexes throughout the rodent brain^28^ by showing that these mechanisms also occur in human dlPFC neurons from patients with cocaine use disorder.

### Associating genetic risk for human disease with the cocaine-regulated brown module

To investigate if genetic disease risk may affect this module, we intersected the brown module genes with a large repository of single nucleotide polymorphisms with genetic risk catalogued by the National Human Genome Research Institute (NHGRI). We found that the brown module was strongly overrepresented with genes associated with genetic risk for disease (*P* = 6.3 × 10^−14^, Odds Ratio = 2[1.7–2.4]), as were the brown hub genes specifically (*P* = 6.1 × 10^−6^, Odds Ratio = 3.6[2.0–6.4]). These hubs include genes associated with body-mass index and obesity related traits (*GNB1*, *PPP1CB*, *RCAN2*, *RSBN1*, *PSD3*, *TOMM20*)^34, 35^; anorexia nervosa (*PPP3CA*)^36^; bipolar disorder (*MARCKS*, *BASP-1*)^37, 38^; and schizophrenia (*GPM6A*)^39^. Since the reward circuitry is central to both addictive and feeding behaviors and several hub genes were associated with BMI and obesity, we specifically tested if BMI and obesity genes were enriched in the brown module. We note that both the overall module (*P* = 0.02, Odds Ratio = 1.9[1.3–2.7]) and its hubs (*P* = 0.003, Odds Ratio = 4.1[1.6–9.2]) are overrepresented for BMI and obesity-related genes. As a negative control, we found that genes associated with height (a phenotype with a similar number of reported GWAS genes as obesity and BMI, but with no apparent relation to addictive phenotypes) were not enriched in the brown module (P > 0.05). This analysis suggests that cocaine use disorder may partly share a molecular etiology with a number of chronic syndromes involving dysfunction in brain reward circuits and identifies several target genes that may mediate this process.

## Discussion

In this study, we performed total RNA-sequencing on post-mortem dlPFC neuronal nuclei from humans with cocaine use disorder and healthy controls. It is important to note that one limitation of our study is our inability to distinguish between the chronic and acute effects of cocaine, since the cocaine use disorder patients were chronic cocaine users who died with cocaine in their system. More work is needed to determine how lethal doses of cocaine alone alter transcription. Still, our dataset represents an important resource for the neurosciences beyond drug addiction, as there are few, if any, studies that have performed total RNA-sequencing on isolated human post-mortem neurons. Given the cell type-specificity of our sequencing, our data can be used by others to infer the expression of specific coding and non-coding transcripts in human post-mortem (in particular, cortical) neurons (See Supplementary Table 2). This will become critical as the diverse functions of non-coding RNAs continue to be explored in the context of translational neuroscience. As many non-coding RNAs are only present in human neurons, our dataset provides an important resource for others to evaluate expression levels of target transcripts specifically in human neurons. The same is true of cell type-specific protein-coding gene expression, as our experiments captured mainly protein coding transcripts as well. The present study focused on males, because of the preponderance of male samples available in our cocaine brain bank (see Methods). It will be important in future studies to extend the approach used here to females as well as to other brain regions.

The differential expression signature that we identified between cocaine addicts and control cases validates many known protein-coding transcripts that have roles in drug addiction and neural plasticity such as *c-Fos* and *c-Jun* and identifies many new coding and non-coding transcripts for further investigation. Furthermore, we provided a novel network-based mechanism for the function of *c-JUN* up-regulation following exposure to cocaine and identify the brown module as one important mediator of the cocaine-exposed phenotype. Since *c-JUN* and other AP1 factors have been previously identified in animal models as mediators of cocaine induced plasticity, our study provides an important step in validating the translation of that basic research to human studies aimed at treating addiction. It is worth noting that *FosB*, which has been strongly implicated in rodent addiction models^31^ did not appear as significantly regulated in our differential expression or network analyses. This may not be surprising since the *FosB* product implicated in addiction, *∆FosB*, accumulates in brain reward regions in response to chronic drug exposure due to the intrinsic stability of the protein, with its mRNA not showing stable induction^40^. Importantly, such accumulation of *∆FosB* has been observed in human nucleus accumbens in cocaine and opiate human addicts^41, 42^. Whether it also accumulates in human dlPFC remains unknown.

Our network analysis also pointed toward the possibility that the transcriptional signature we identified in the brown module may be affected in a number of chronic conditions. The intersection of module hubs with genetic risk factors for feeding-related traits (obesity and body-mass index) is particularly striking given the emerging functional imaging evidence that shows metabolic and functional alterations in dlPFC of obese humans. Our molecular data is consistent with these functional studies and provide support for the theory that obesity may involve dysfunction in the dlPFC component of the reward circuitry^43, 44^. Our results raise the possibility that integrative network analyses of cell-type specific human brain samples can reveal new biological phenomena and provide novel insight into psychiatric disease that complements well-established clinical paradigms.

Our finding of robust IEG activation is in line with previous rodent studies of cocaine induced plasticity in PFC, and supports the notion that transcriptional alterations in dlPFC neurons of human addicts may alter long-term neuroplasticity through the downstream actions of these IEG pathways. In particular, gene ontology analysis of the brown module identified small GTPase signaling (*RAP2A*, *RAB1B*, *KRAS*, *RAN*) which is strongly implicated in synaptic regulation, as most significantly enriched. This finding shows that we are able to detect changes in transcripts whose protein products ultimately regulate synapses through nuclear sequencing. Small GTPase signaling has been shown to be critical for the induction of cocaine-induced plasticity in nucleus accumbens medium spiny neurons. A recent study has shown that the translocation of small GTPases from the nucleus to the synapse alters the structure of dendritic spines, and ultimately the physiology of the neuron^45^. These studies would suggest that alterations of the brown module in dlPFC neurons may underlie changes in synaptic structural plasticity in human addicts, and establishes this pathway as a potential target for the generation of future therapeutics for substance use disorders. From the transcriptional signature we identified, it may be warranted to explore the potential of altering the brown module for the generation of therapies based on cell type-specific RNA-sequencing in human brain.

## Methods

### Post-Mortem Brain Samples

All methods were carried out in accordance with guidelines and regulations established at Icahn School of Medicine at Mount Sinai for use of human samples. Experimental protocols were approved by Icahn School of Medicine at Mount Sinai. Informed consent was obtained from all patients or families prior to autopsy in accordance with standards established at University of Miami Miller School of Medicine. Brain specimens were obtained during routine autopsy from unaffected control subjects who died suddenly and from cocaine-related deaths. Medicolegal investigations and certifications of the cocaine intoxication deaths were conducted by forensic pathologists to determine the cause and manner of death (Supplementary Table 1). The circumstances of death and blood and brain toxicology were reviewed to identify sudden deaths due to the toxic effects of chronic cocaine abuse in persons who met criteria for cocaine use disorder (n = 19). Drug-free age-matched control subjects (n = 17) were selected from homicides, accidental or natural deaths that had negative urine screens for all common drugs in decedents with no history of licit or illicit drug use before death. Cocaethylene is a measure of concurrent cocaine and alcohol use. Alcohol, cocaethyelene and nicotine were measured in post-mortem blood to confirm recent use. PMI (post-mortem interval), RIN, age, and race are provided and do not significantly differ between cases and controls (Table 1).

Frozen human dlPFC tissue was obtained from the University of Miami Brain Bank in accordance with their institutional ethics board. Anatomic specimens were sampled from the middle frontal gyrus at the lateral part of Brodmann area 46. The brain samples were matched for sex (male), age, race, smoking history, PMI and brain pH to normalize factors that can affect brain RNA quality.

This study used male samples only because of the preponderance of males in the available brain bank. Cocaine use still predominates in males overall and the incidence of cocaine intoxication deaths follows this pattern. Also, the rates of polydrug abuse and psychiatric comorbidity are higher among females^46^ further excluding females based on the criteria for subject inclusion for the present study. Blood Alcohol Contents are provided in Supplementary Table 1.

### Fluorescence-Activated Cell Sorting (FACS) of Neuronal Nuclei

Neuronal nuclei were sorted on a BD FACS Aria II using a novel method for isolation of total RNA from human post-mortem tissue. Briefly, the brain tissue was pulverized on dry ice and 70–100 mg of frozen tissue was used per sample for FACS. The frozen tissue was then dounced-homogenized on ice with nuclear lysis buffers, and the subsequent suspension was centrifuged in a sucrose gradient at 24,400 K RPM at 4 °C using a swing bucket rotor to isolate nuclei. The resulting pellets were resuspended in PBS containing PE-conjugated mouse anti-NeuN antibody (MilliMark Anti-NeuN-PE Antibody Clone A60; Millipore, USA). The samples were rotated in the dark at 4 °C for 15 minutes before FACS. DAPI was used to isolate nuclei prior to gating for NeuN-PE. 70,000 neuronal nuclei were sorted directly into Trizol LS (Life Technologies), vortexed and immediately flash frozen on dry ice until RNA extraction.

### RNA Extraction and Total RNA Library Preparation

To isolate RNA from the samples in Trizol LS, we used the Zymo Directzol RNA miniprep kit (Zymo Research; R2050). We performed DNAse treatment on all samples to ensure removal of genomic DNA before proceeding with library preparation. RNA was analyzed for size distribution and quantity using Bioanalzyer 2000 (Agilent Technologies, USA). Since we removed cytoplasmic contents in the process of sorting nuclei, we observed a small but consistent percentage of ribosomal RNA.

We used the Clontech SMARTer Stranded Total RNA-seq library preparation kit to create indexed libraries from 10 ng of total nuclear RNA from each sample for sequencing (Clontech/Takara; 634839). All libraries were generated simultaneously using a multiplexed library preparation strategy to ensure no variation in processing. Resulting libraries were analyzed for size distribution and quantity using PCR, Agilent Bioanalyzer and Tapestation prior to sequencing.

### Total RNA-Sequencing

Molar equivalent libraries were pooled in 4 groups of 10 for multiplexed sequencing across one flow cell on the Illumina Hiseq-2000. Each pool contained equal number of cases and control samples. Each pool was also sequenced on two different lanes to reduce the effect of lane on the data. Using V4 chemistry, we performed 2X125 BP PE RNA-sequencing and obtained >100,000,000 reads per sample. The raw FASTQ sequencing data will be made publically available in the Gene Expression Omnibus (GEO) upon publication. Both DAVID and Enrichr were used in our gene investigations since they offer complementary analytical features.

### Differential Gene Expression Analysis

STAR was used to align the FASTQ files to the standard human reference genome (hg19). Reads were quantified with the featureCounts tool in the Subread package and collated into a count matrix. Transcripts with fewer than 1 count per million in more than 10 samples were filtered out and the remaining transcripts were transformed to variance-stabilized log-counts per million (log-CPM). For these transcripts, we investigated the potential influence of covariates on their expression. For each covariate, ANOVA p-values were calculated for all genes. If the covariate did not have a widespread effect on gene expression, these p-values are expected to be uniformly distributed. For each covariate, the Kolmogorov-Smirnov test was used to estimate if the associated ANOVA p-values deviated significantly from a uniform distribution. This analysis revealed that RIN and PMI had strong widespread effects on gene expression (p < 0.01) (Supplementary Figure 1). Since the effects of one covariate could mask the effects of others, we used VariancePartition to decompose gene expression variation into the variance attributable to each covariate^47^. We calculated the variance explained by each covariate for each gene, which confirmed the widespread effect of RIN and PMI on gene expression variability (Supplementary Figure 2). Though the effect of age and race was less widespread, this analysis also showed that these covariates strongly influence the expression of many transcripts. Based upon these two analyses, we determined that RIN, PMI, age, and race needed to be accounted for when modeling differential expression. Voom was used to identify differentially expressed genes, with RIN, PMI, age, and race included in the model as covariates. Surrogate variable analysis was also used to identify “hidden” covariates, and these surrogate variables were also included in the linear model for differential expression.

### Calculating Gene Coexpression Networks and Their Association with Chronic Cocaine Use

Weighted gene coexpression network analysis was used to identify gene modules in the normalized and corrected log-CPM expression matrix. Specifically, linear relationships between all transcripts were described by their pairwise Pearson correlation. The adjacency matrix of the gene expression graph was described as the Pearson correlation raised to a positive power, β, which will be then related to a “scale-free” power law connectivity distribution p(k) by regressing log(p(k)) on log(k). For this analysis, a β value of 9 was selected (Supplementary Figure 3). The adjacency matrix was further quadratically transformed to take into account nearest-neighbor links via topological overlap matrix (TOM), which has been shown to better depict biological interactions and thus segregate relevant modules. A hierarchical clustering method then was used to group genes based upon their topological overlap, and these groups were segregated into modules by implementing the Dynamic Tree Cut algorithm, with a minimum module size of 30. Colors were used to identify coexpression modules, with the grey module representing genes that do not segregate into any particular module. Finally, the robustness and reproducibility of each module was estimated by repeatedly splitting the data into training and test sets and calculating a module preservation score between the resulting networks. Averaging the preservation statistics across multiple iterations of random data splitting resulted in a robustness measure for each module. Module enrichments for differentially expressed genes and GWAS genes were calculated with Fisher’s exact test, and module hub genes were defined as the 10% most (intra)connected members within a module. Lastly, the first principal component (eigengene) of each module was calculated and associations between module eigengenes and status were calculated using the Kruskal-Wallis test.

## Electronic supplementary material

Supplementary Information

Supplementary Table 1

Supplementary Table 2
