# Supplementary material for: Gene Network Dysregulation in Dorsolateral Prefrontal Cortex Neurons of Humans with Cocaine Use Disorder
Source: Sci Rep. 2017 Jul 14;7:5412. doi: 10.1038/s41598-017-05720-3 (PMC5511210; doi:10.1038/s41598-017-05720-3)
Supplement: Supplementary file 1 — Supplementary Information [file 41598_2017_5720_MOESM1_ESM.doc]

**Supplementary Information**

**Title:** Gene Network Dysregulation in Dorsolateral Prefrontal Cortex Neurons of Humans with Cocaine Use Disorder

**Authors:** Efrain A. Ribeiro*^1^, Joseph R. Scarpa*^2^, Susanna P. Garamszegi^3^, Andrew Kasarskis^2^, Deborah C. Mash^3^, Eric J. Nestler^1^

**Affiliations:** ^1^Fishberg Department of Neuroscience, Friedman Brain Institute, Icahn School of Medicine at Mount Sinai, New York, NY, USA; ^2^Department of Genetics and Genomic Sciences, Icahn Institute for Genomics and Multi-Scale Biology, Icahn School of Medicine at Mount Sinai, New York, NY, USA; ^3^Department of Neurology, University of Miami Miller School of Medicine, Miami, Florida, USA.


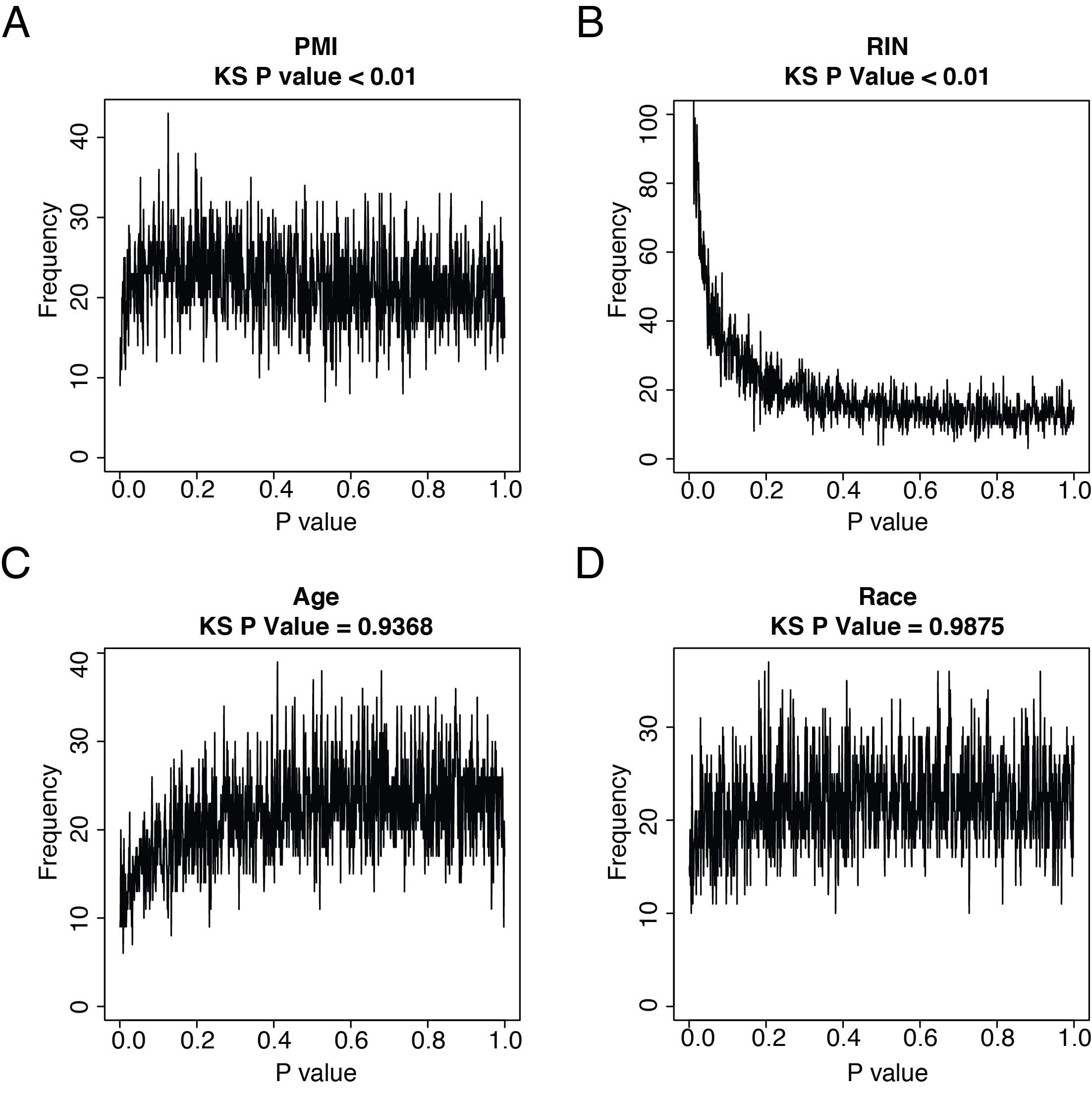


**Supplementary Figure 1:** The distribution of ANOVA p-values reflects the association between each covariate and gene expression and are plotted her for each covariate. Kolmogorov-Smirnov test p-values are also reported in the header, estimating the probability that the ANOVA p-values were drawn from a uniform distribution.


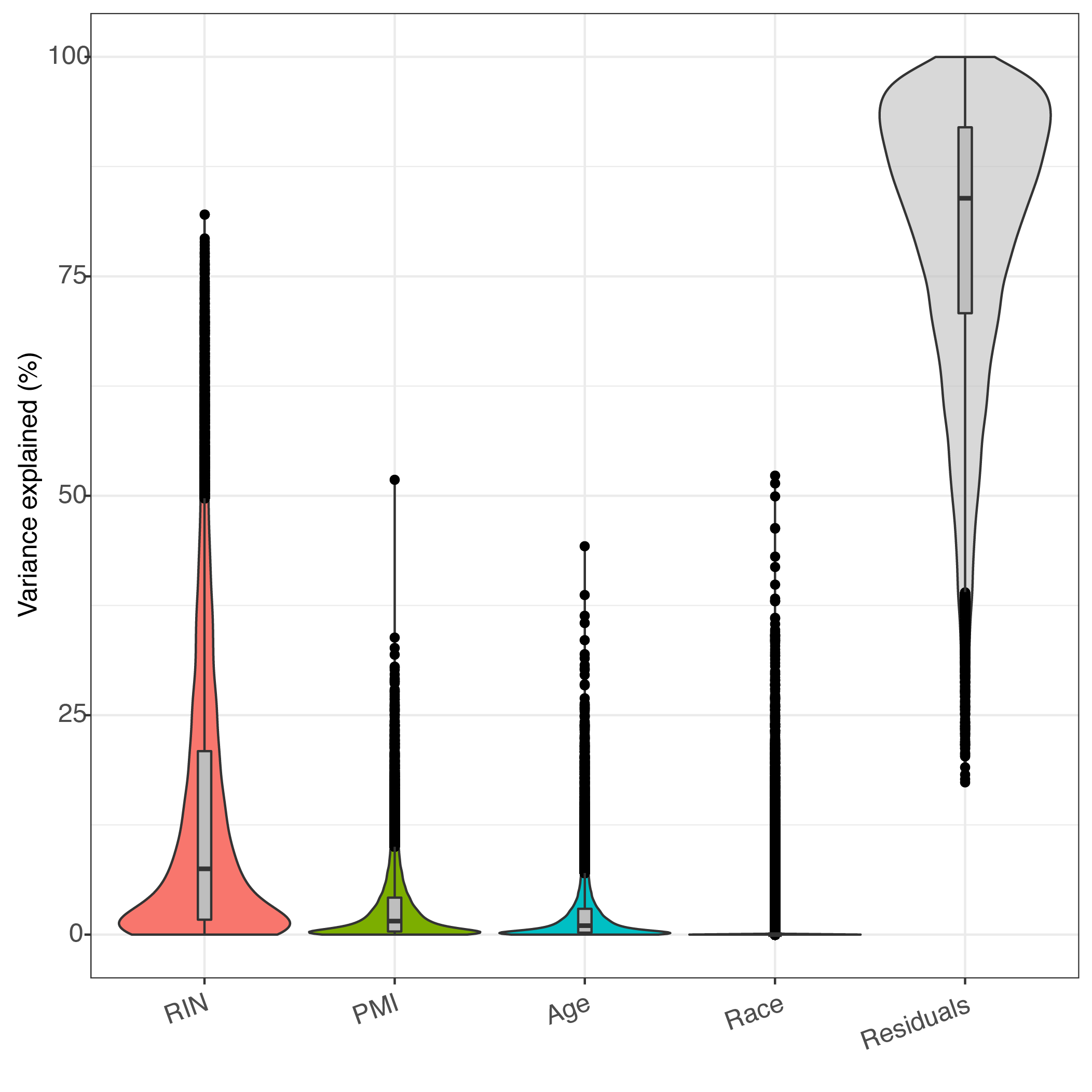


**Supplementary Figure 2:** The percent variance explained for each gene by each covariate shows that RIN, PMI, age, and race have a strong effect on the expression of many genes. All four covariates were adjusted for in downstream analyses.


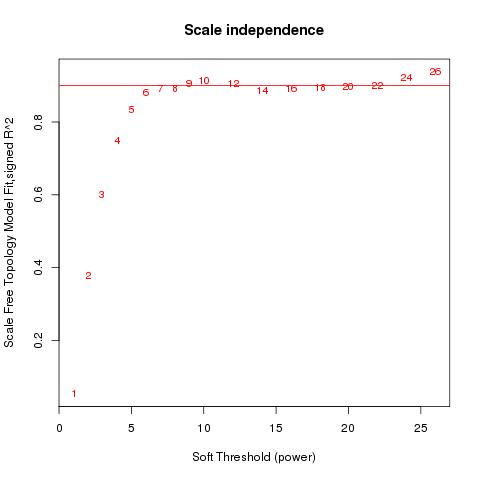


**Supplementary Figure 3:** The scale-free topology fit index improves as the Pearson correlation matrix is raised to successively higher powers (β). We selected β of 9, since it is the smallest coefficient with a scale-free R^2^ ≥ 0.9 and the fit index plateaus at greater powers.

**
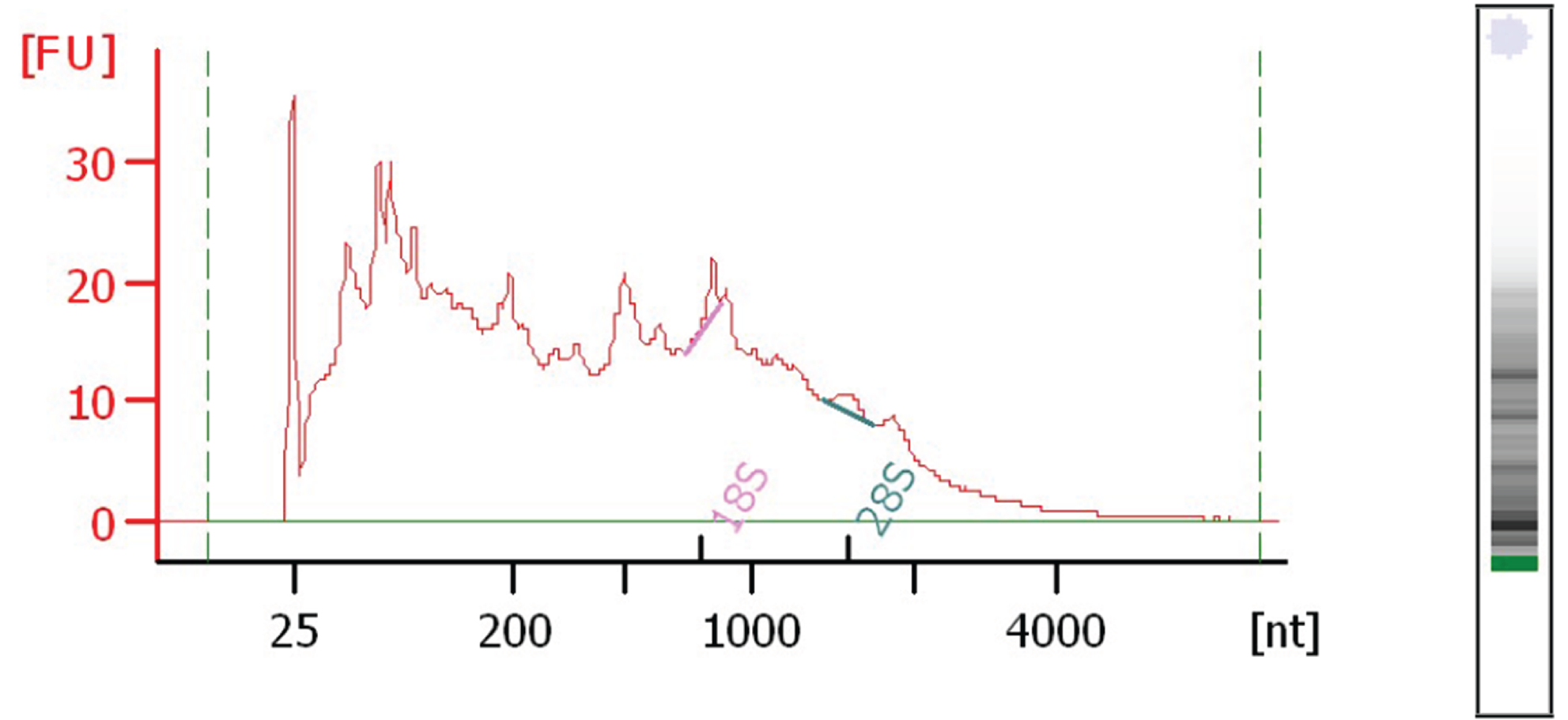
Supplementary Figure 4:** Representative RNA bioanalzyer trace showing wide distribution of transcript lengths captured. Note that there are no large peaks present at the 18S or 28S bands as would be expected in a whole tissue extract.
